# The dilution effect and the importance of selecting the right internal control genes for RT-qPCR: a paradigmatic approach in fetal sheep

**DOI:** 10.1186/s13104-015-0973-7

**Published:** 2015-02-27

**Authors:** Huaisheng Xu, Massimo Bionaz, Deborah M Sloboda, Loreen Ehrlich, Shaofu Li, John P Newnham, Joachim W Dudenhausen, Wolfgang Henrich, Andreas Plagemann, John RG Challis, Thorsten Braun

**Affiliations:** Departments of Obstetrics and Division of Experimental Obstetrics, Charité - University Berlin, Augustenburger Platz 1, Berlin, Germany; Departments of Obstetrics and Gynecology, Linyi People’s Hospital, Shandong, China; Animal and Rangeland Sciences, Oregon State University, Corvallis, USA; Departments of Biochemistry and Biomedical Sciences, Obstetrics & Gynecology and Pediatrics, McMaster University, Hamilton, Canada; School of Women’s and Infants’ Health, King Edward Memorial Hospital, The University of Western Australia, and Women and Infants Research Foundation of Western Australia, Perth, Australia; Departments of Physiology, Obstetrics and Gynecology, University of Toronto, Toronto, Canada; Faculty of Health Sciences, Simon Fraser University, Vancouver, Canada

**Keywords:** Dexamethasone, Sheep, Fetal liver, geNorm, Co-regulation, Internal control gene, *RNA18S*, RPLP0, HPRT1, PPIA, TUBB, ACTB, *RNA28S*

## Abstract

**Background:**

The key to understanding changes in gene expression levels using reverse transcription real-time quantitative polymerase chain reaction (RT-qPCR) relies on the ability to rationalize the technique using internal control genes (ICGs). However, the use of ICGs has become increasingly problematic given that any genes, including housekeeping genes, thought to be stable across different tissue types, ages and treatment protocols, can be regulated at transcriptomic level. Our interest in prenatal glucocorticoid (GC) effects on fetal growth has resulted in our investigation of suitable ICGs relevant in this model. The usefulness of *RNA18S*, *ACTB*, *HPRT1*, *RPLP0*, *PPIA* and *TUBB* as ICGs was analyzed according to effects of early dexamethasone (DEX) treatment, gender, and gestational age by two approaches: (1) the classical approach where raw (i.e., not normalized) RT-qPCR data of tested ICGs were statistically analyzed and the best ICG selected based on absence of any significant effect; (2) used of published algorithms. For the latter the geNorm Visual Basic application was mainly used, but data were also analyzed by Normfinder and Bestkeeper. In order to account for confounding effects on the geNorm analysis due to co-regulation among ICGs tested, network analysis was performed using Ingenuity Pathway Analysis software. The expression of *RNA18S*, the most abundant transcript, and correlation of ICGs with *RNA18S,* total RNA, and liver-specific genes were also performed to assess potential dilution effect of raw RT-qPCR data. The effect of the two approaches used to select the best ICG(s) was compared by normalization of *NR3C1* (glucocorticoid receptor) mRNA expression, as an example for a target gene.

**Results:**

Raw RT-qPCR data of all the tested ICGs was significantly reduced across gestation. *TUBB* was the only ICG that was affected by DEX treatment. Using approach (1) all tested ICGs would have been rejected because they would initially appear as not reliable for normalization. However, geNorm analysis (approach 2) of the ICGs indicated that the geometrical mean of *PPIA*, *HPRT1*, *RNA18S* and *RPLPO* can be considered a reliable approach for normalization of target genes in both control and DEX treated groups. Different subset of ICGs were tested for normalization of *NR3C1* expression and, despite the overall pattern of the mean was not extremely different, the statistical analysis uncovered a significant influence of the use of different normalization approaches on the expression of the target gene. We observed a decrease of total RNA through gestation, a lower decrease in raw RT-qPCR data of the two rRNA measured compared to ICGs, and a positive correlation between raw RT-qPCR data of ICGs and total RNA. Based on the same amount of total RNA to performed RT-qPCR analysis, those data indicated that other mRNA might have had a large increase in expression and, as consequence, had artificially diluted the stably expressed genes, such as ICGs. This point was demonstrated by a significant negative correlation of raw RT-qPCR data between ICGs and liver-specific genes.

**Conclusion:**

The study confirmed the necessity of assessing multiple ICGs using algorithms in order to obtain a reliable normalization of RT-qPCR data. Our data indicated that the use of the geometrical mean of *PPIA*, *HPRT1*, *RNA18S* and *RPLPO* can provide a reliable normalization for the proposed study. Furthermore, the dilution effect observed support the unreliability of the classical approach to test ICGs. Finally, the observed change in the composition of RNA species through time reveals the limitation of the use of ICGs to normalize RT-qPCR data, especially if absolute quantification is required.

**Electronic supplementary material:**

The online version of this article (doi:10.1186/s13104-015-0973-7) contains supplementary material, which is available to authorized users.

## Background

Quantitative reverse transcription real time polymerase chain reaction (RT-qPCR) is the most sensitive and accurate method to measure mRNA gene expression. Accurate normalization is critical in accounting for quantity of input RNA, varying amounts of cDNA input, sample loss during handling, and activity and variation in the kinetics of reverse-transcription enzyme during reaction [1,2]. A reliable normalization may be even more important when the experiment aims to evaluate low-fold changes of a target gene [3].

Among several proposed method of normalization, including total RNA, genomic DNA, and spike-in of an external artificial reference [4], the use of internal control genes (ICGs) appear to be the most accurate [5]. Validated ICG are essential for unbiased interpretation of RT-qPCR given the vast number of confounding factors that can affect the transcription of any gene [4]. Classically, housekeeping genes (HKG) were used as ICGs because it was thought that they expression is stable across different tissue types, ages, and treatments; however, several studies revealed that also the expression of HKG is under active regulation [4]. This was suggested by some studies where it was shown that expression of HKG may vary as a result of neoplastic growth, hypoxia or other experimental treatments [6-9]. Among classic HKG, beta-actin (*ACTB*) mRNA expression for example is increased by a maximum of 4-fold in fibroblasts during 8 hrs of serum stimulation [8]. *ACTB* and cyclophilin A (*PPIA*) expression in human tumor cell lines also varied widely after hypoxia treatment [6]. The expression of ribosomal protein, large, P0 (*RPLP0*) was significantly changed after hindlimb suspension intervention in rat tendon and muscle [10]. With lipopolysaccharide stimulation, the expression level of hypoxanthine phosphoribosyltransferase 1 (*HPRT1*) and tubulin, beta (*TUBB*) increased in alveolar macrophages from chronic obstructive pulmonary disease patients [11]. All the above clearly demonstrated that the use of classic HKG for RT-qPCR normalization without any assessment is highly unreliable. In addition, they also indicated that the ICGs do not need to be HKG. Therefore, there was a need of a new approach for testing the reliability of ICGs for each experiment. In order to evaluate the reliability of ICGs for RT-qPCR normalization several algorithms have been developed; those include geNorm Visual Basic (geNorm v3.5) [2], Bestkeeper [12], and NormFinder [13]. Studies dealing with very different conditions, including tissue/organ development, have successfully used geNorm in selecting reliable ICGs [14-22], a method that has been statistically validated [23]. Those studies have shown the importance of using the geometric mean of at least two stably expressed ICGs. In addition, some of those studies have also demonstrated the detrimental effect of improper selection of ICGs on RT-qPCR results of target genes of interest. The geNorm program assesses the stability of gene expression between candidate reference genes by performing a pair-wise comparison of the expression ratio. The fundamental rationale of the geNorm algorithm is that the higher the stability of expression ratio between two non-coregulated candidate genes across the samples, the higher the likelihood that those are stably expressed; thus, highly reliable ICGs. Therefore, it is critical prior to geNorm analysis to verify the absence of co-regulation among potential ICGs which otherwise would bias geNorm results [24].

Despite the critical importance of proper selection and evaluation of ICGs, these are often chosen based upon previously scientific publications rather than upon empirical data. Furthermore, the evaluation of ICGs reliability is performed by analyzing changes in the raw RT-qPCR in respect to the experimental conditions (e.g., gestational age, sex or the effects of a special treatment). This approach, however does not take the accumulation of errors and variations in each single sample during the analytical procedure or the dilution effect of stably expressed genes due to large increase in expression of very abundant transcripts into account [1].

Prenatal synthetic glucocorticoid (GC) treatment is commonly used in the management of women at risk of early preterm birth and has succeeded in reducing neonatal mortality and morbidity [25]. Despite these benefits, GC treatment has also been associated with a decrease in birth weight and alterations in glucose homeostasis and hypothalamic–pituitary–adrenal (HPA) axis function and fetal programming [26-28]. Although the exact mechanisms underlying those responses are unknown, fetal hepatic development may play an important role [29]. The liver, largest of the body’s organs, plays an important role in coordinating metabolic homeostasis, nutrient processing and detoxification [30]. Many studies suggest the liver to be a target organ of GC treatment and partly responsible for fetal growth restriction [26,31,32] and gene expression profiling might help dissecting underlying processes. Early maternal GC treatment (dexamethasone, DEX) is used in suspected cases of congenital adrenal hyperplasia to protect female fetuses from virilization [33]. We have shown that DEX treatment analogous to that used in human subjects in the first third of pregnancy, resulted in profound changes in fetal HPA axis development which persisted during postnatal [26,34].

In order to study the effect of early DEX treatment on liver gene expression using RT-qPCR it is essential to have reliable ICGs. In previous studies involving sheep under different experimental conditions, internal references such as 18S ribosomal RNA (*RNA18S*), *ACTB*, *HPRT1*, *RPLP0*, *PPIA* and *TUBB* have been widely used to investigate a range of target genes. All those ICGs were chosen in these studies based solely on previous publications in different experimental models [35-38] and not a thorough evaluation of reliability was performed. There are no previous data on the selection of reliable ICGs in fetal sheep liver which are independent of gestational age, sex and not regulated by GC treatment.

Within this framework we set out to identify reliable ICGs to be used for normalize RT-qPCR data from the experiment that investigate the effects of early DEX treatment on the fetal sheep liver transcriptome during gestation. For this we have tested the 6 ICGs previously used in sheep with the aim to test the expression stability, identify the most reliable, and uncover the number of ICGs that should be used for accurate RT-qPCR normalization. We evaluated the reliability of ICGs following two approaches: (1) we performed a statistical analysis of the raw RT-qPCR data to assess effect of DEX, gestation, sex, and their interaction (i.e., “the classical analysis”) and (2) by testing the stability and reliability of the normalization by using algorithms, particularly geNorm Visual Basic application (“geNorm analysis”). The effect on normalization based on the two approaches and different subsets of ICGs was assessed on the RT-qPCR data of glucocorticoid receptor (*NR3C1*).

## Results

### Approach 1: Expression levels of ICGs, “the classical analysis”

**Figure 1:**
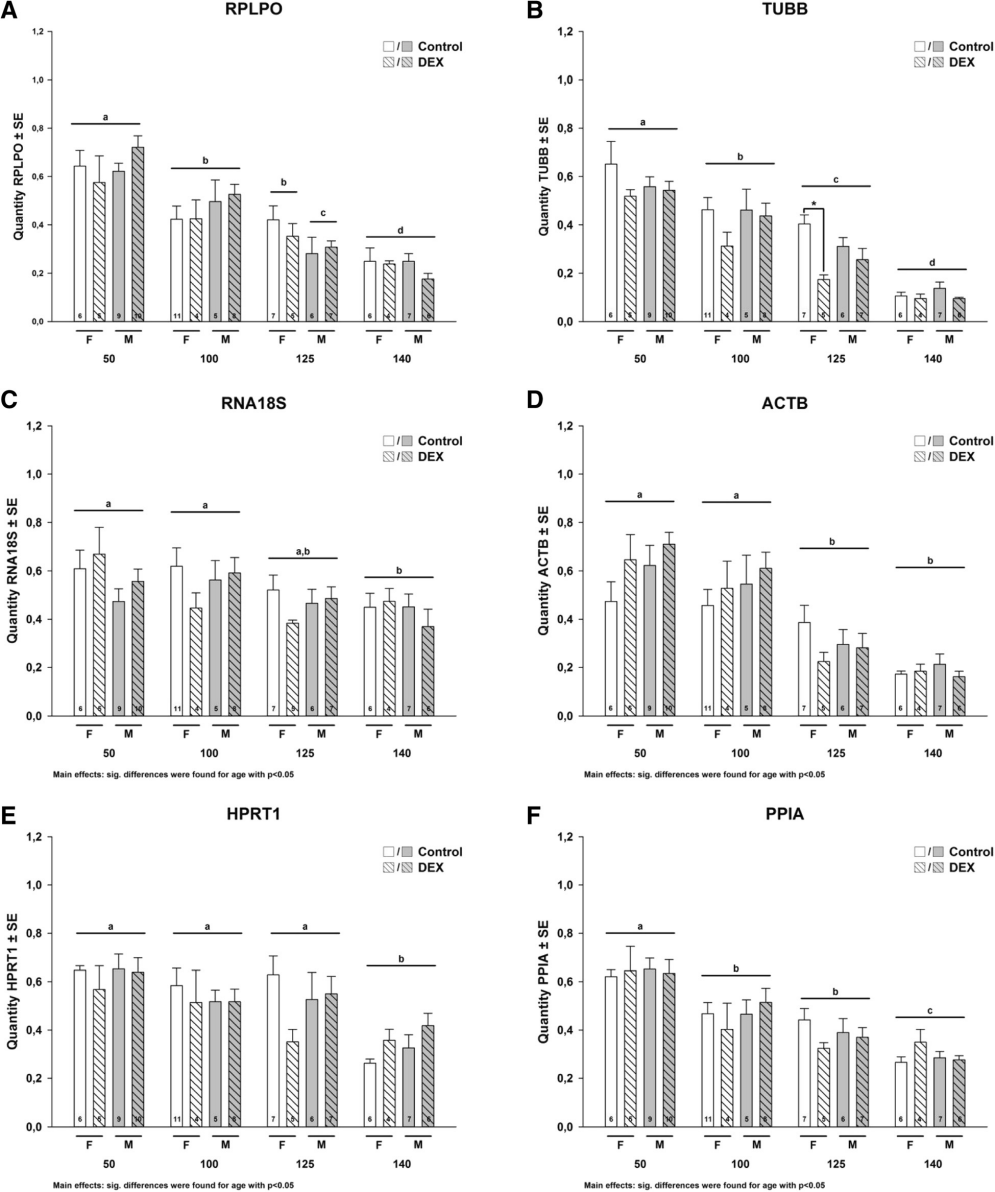
**A-F: The effect of early DEX treatment on the raw RT-qPCR data of 6 ICGs in fetal sheep liver.** Data were analyzed by MANOVA with treatment, gender and days of gestation as factors, followed by a pairwise comparison (Holm’s Sidak) when main effects were p < 0.05. Different letters indicate significant differences in day of gestation and stars significant differences in treatment. n = numbers of animals included in the study.

Raw RT-qPCR data of all ICGs tested significantly (p < 0.05) decreased due to gestation between 50 and 140dG in all tested ICGs; however patterns were slightly different between ICGs (Figure 1). *RPLPO*, *TUBB* and *PPIA* significantly decreased between 50 and 140dG (Figure 1A, B, F), whereas *ACTB* levels stayed stable between 50 and 100dG (p > 0.05), but significantly decreased between 100 and 140dG (Figure 1C, D). *HPRT1* and *RNA18S* levels stayed stable between 50 and 125dG and significantly decreased at 140dG (Figure 1E). Compared to control, *TUBB* raw RT-qPCR data was significantly lower at 125dG in female treated with DEX; raw RT-qPCR data of the other ICGs were not affected by DEX treatment (Figure 1). Gender differences were only found in *RPLPO* at 125dG, with significantly higher expression levels in females as compared to males (Figure 1A). In summary, all ICGs tested were affected by gestation and some by DEX and/or gender. Among the tested ICGs the raw RT-qPCR of *HPRT1* and *RNA18S* were the least affected by the tested conditions; thus, can be considered the best choice for normalization of RT-qPCR of target genes among the 6 tested ICGs.

### Approach 2: Determination of ICGs expression stability using algorithms

**Figure 2:**
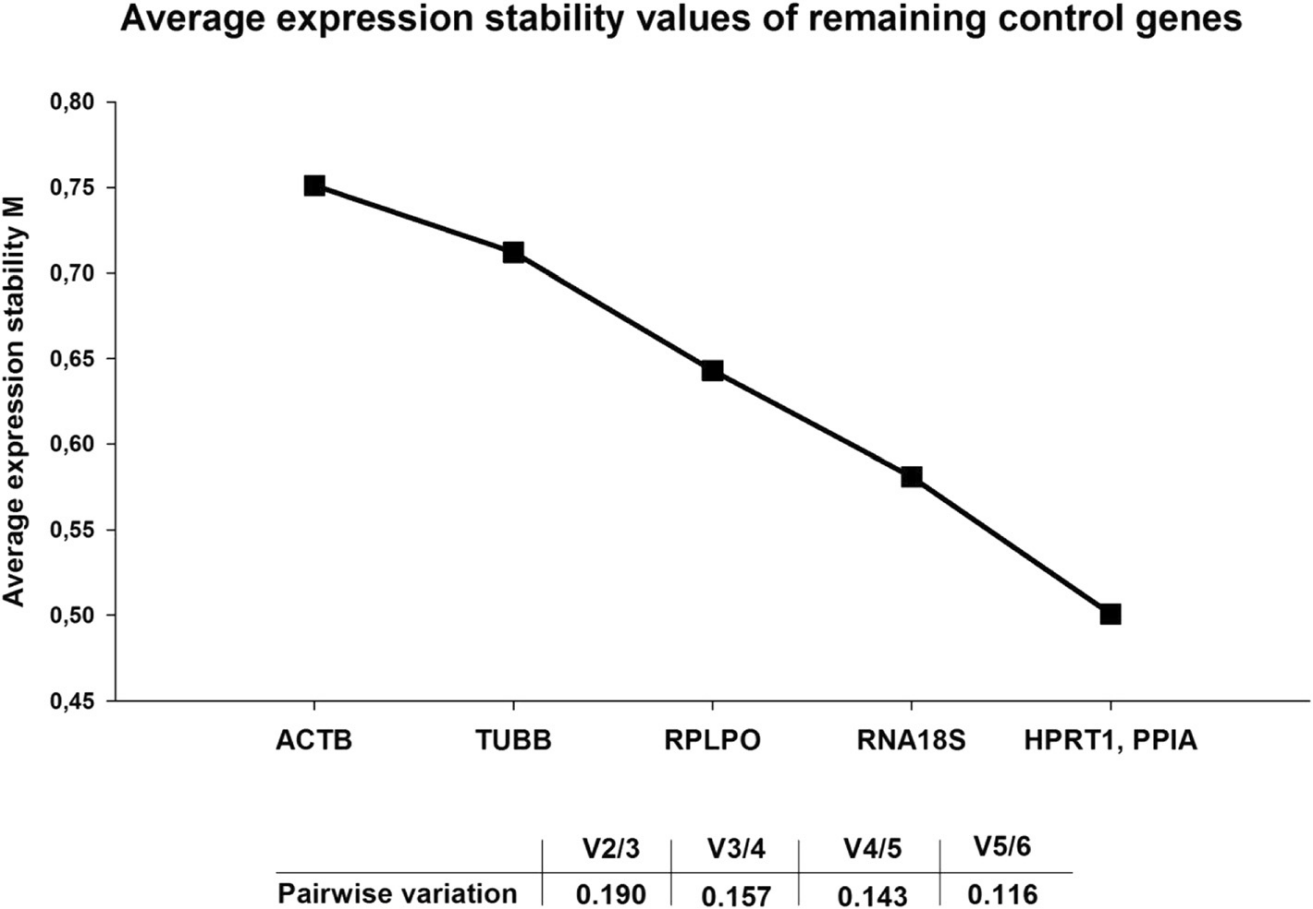
**Average expression stability values of tested potential internal control genes (ICGs).** Average expression stability values (M) of remaining ICGs and determination of the optimal number of genes for normalization performed by geNorm, measured in n = 106 fetal sheep liver samples. The x axis indicates the ranking of the ICGs from least (left) to most (right) stable. The pairwise variation indicates the increase in normalization factor reliability by adding additional less stable ICGs.

The raw RT-qPCR data of the 6 tested ICGs were analyzed with geNorm, Bestkeeper, and Normfinder. The rank from the most to the least reliable ICG was similar between the three algorithms (Table 1), with PPIA being one of the two most reliable ICGs among the ones tested. In geNorm the lowest M value indicates ICGs with the most stable expression. In the overall analyses, stepwise elimination of successive genes showed that *PPIA* and *HPRT1* were the most stable ICGs across gestation followed by *RNA18S* and *RPLPO* (Figure 2). The determination of the optimal number of ICGs for normalization is performed by geNorm by calculating the pairwise variation (V-value) of adding the subsequent more reliable gene. A V-value below 0.15, cut-off reported by Vandesompele *et al.* [2], was obtained by adding the 5th more reliable gene (V4/5); however, the addition of the 4th more reliable gene (i.e., V3/4) had a V-value of 0.157, which is similar to V4/5 (Figure 2). Based on this observation and based also on practicality, we deemed that the use of 4 most reliable ICGs among the one tested, that is *PPIA*, *HPRT1, RNA18S* and *RPLPO,* can provide a trustworthy normalization factor.

**Table 1:** **Comparison of the expression stability of 6 reference genes in fetal sheep liver as calculated by geNorm, Normfinder and BestKeeper**

**Rank**	**geNorm**		**NormFinder**		**BestKeeper**		**BestKeeper**	
	**Gene**	**Stability**	**Gene**	**Stability**	**Gene**	Stability*	**Gene**	Stability**
**1**	*PPIA*	0.50	*PPIA*	0.188	*HPRT1*	2.17	*RNA18S*	27.2
**2**	*HPRT1*	0.50	*HPRT1*	0.326	*PPIA*	2.43	*PPIA*	32.2
**3**	*RNA18S*	0.58	*RPLPO*	0.349	*RNA18S*	3.26	*HPRT1*	33.3
**4**	*RPLPO*	0.64	*RNA18S*	0.410	*RPLPO*	3.60	*RPLPO*	40.7
**5**	*TUBB*	0.71	*TUBB*	0.443	*ACTB*	3.75	*TUBB*	49.1
**6**	*ACTB*	0.75	*ACTB*	0.454	*TUBB*	4.45	*ACTB*	50.0

*% coefficient of variation compared to the mean cycle at the threshold (Ct).

**% coefficient of variation compared to the mean relative abundance (calculated as E^Ctmin-Ctsample^).

geNorm is one of several algorithms available to evaluate ICGs. We have evaluated our potential ICGs also using Bestkeeper and Normfinder (Table 2). There was an overall agreement about the 3 algorithms to rank the best vs. the worst ICGs tested with some small differences, the major one being *RNA18S* as the most reliable ICG in Bestkeeper when considering the calculated raw RT-qPCR data instead of Ct values. The latter is the method originally used to develop Bestkeeper; thus, likely more reliable [12].

**Table 2:** **Pearson correlations between RNA concentration and raw RT-qPCR data of ICGs, rRNAs, and liver-specific genes**

**Gene**	**RNA (ug/ug tissue)**	***IGF1***	***G6PC***
***ACTB***	0.394**	−0.236*	−0.449**
***TUBB***	0.424**	−0.454**	−0.575**
***RPLPO***	0.469**	−0.476**	−0.396**
***RNA18S***	0.219*	**−0.131**	−0.141
***HPRT1***	**0.178**	−0.219*	−0.368**
***PPIA***	0.465**	−0.471**	−0.484**
**All ICG**	0.468**	−0.429**	−0.529**
***RNA28S***	0.077	0.146	−0.01**
***IGF1***	−0.479**		0.340*
***G6PC***	−0.301*	−0.340*	

The best ICGs are highlighted by bold.

**indicates correlation with p < 0.01; *indicates correlation with p < 0.05.

**Figure 3:**
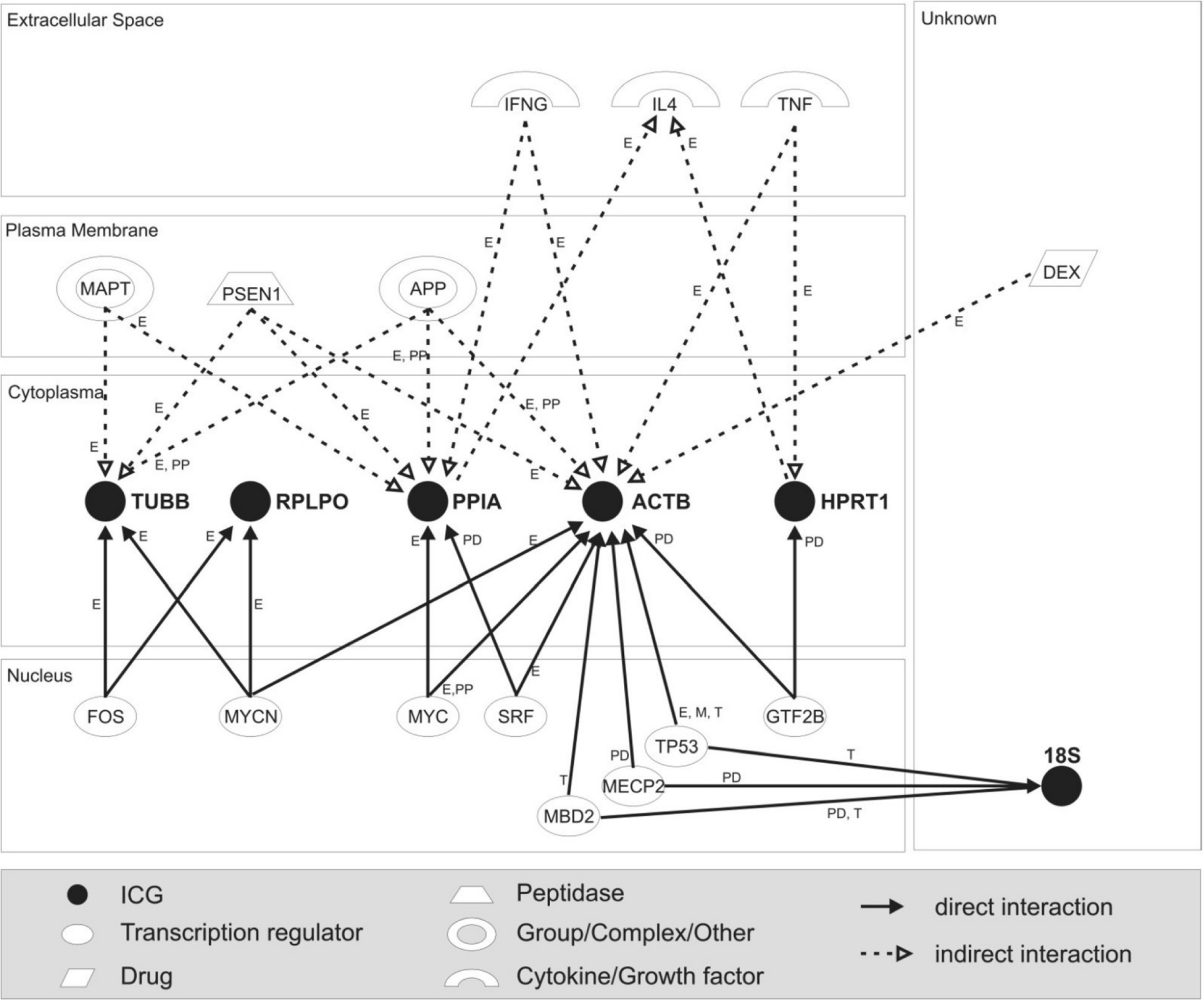
**Co-regulation analysis among 6 potential internal control genes (ICGs).** The network analysis of potential co-regulation among 6 ICGs was performed with Ingenuity Pathway Analysis (IPA). Solid lines indicate direct interactions and dotted lines indirect interactions. Edge labels indicate effects on gene expression (E), protein-DNA interactions (PD), effect on transcription (T) and protein-protein interactions (PP). Arrows indicate the direction of the effect. **ICGs:** *HPRT1* (hypoxanthine phosphoribosyltransferase), *PPIA* (peptidylprolyl isomerase A), *18S* (18S ribosomal rRNA), *ACTB* (beta actin), *RPLPO* (ribosomal protein, large, P0) and *TUBB* (beta-tubulin). **Potential co-regulators:** IFNG (interferon gamma), IL4 (Interleukin 4), TNF (tumor necrosis factor), MAPT (microtubule-associated protein tau), PSEN1 (presenilin 1), APP (amyloid bet (A4) precursor protein), FOS (FBJ murine osteosarcoma viral), MYCN (v-myc cytelomatosis viral oncogene homolog), MYC (v-myx cytelomatosis viral), SRF (serum response factor), NFE2L2 (nuclear factor-like 2), GTF2B (general transcription factor IIB), MBD2 (methyl-CpG binding domain protein 2), MECP2 (methyl CpG binding protein 2), TP53 (tumor protein p53).

The geNorm algorithm is based on the assumption that the tested genes are completely independent; i.e., they are not co-regulated because they do not have up-stream common regulator(s). Therefore, it is essential to verify that the best ICGs are indeed independent. For this, potential co-regulation was analyzed using Ingenuity Pathway Analysis (IPA). The use of IPA revealed a co-regulation (or common up-stream regulator(s)) between *RNA18S* and *ACTB via* tumor protein p53 (TP53), methyl CpG binding protein 2 (MECP2), and methyl-CpG binding domain protein 2 (MBD2) [39-46]. Direct co-regulation has been observed between *TUBB*, *RPLPO* and *ACTB via* v-myc myelocytomatosis viral (MYCN) [47-49] and between *TUBB* and *RPLPO via* FBJ murine osteosarcoma viral (FOS) [49,50]. However, no direct co-regulation has been observed between *HPRT1*, *PPIA*, *RNA18S* and *RPLPO* (Figure 3), the ICGs uncovered by geNorm to be reliable, and the most reliable among the one tested.

### Normalization of a target gene by using best ICG(s) uncovered in approach 1 and 2

**Figure 4:**
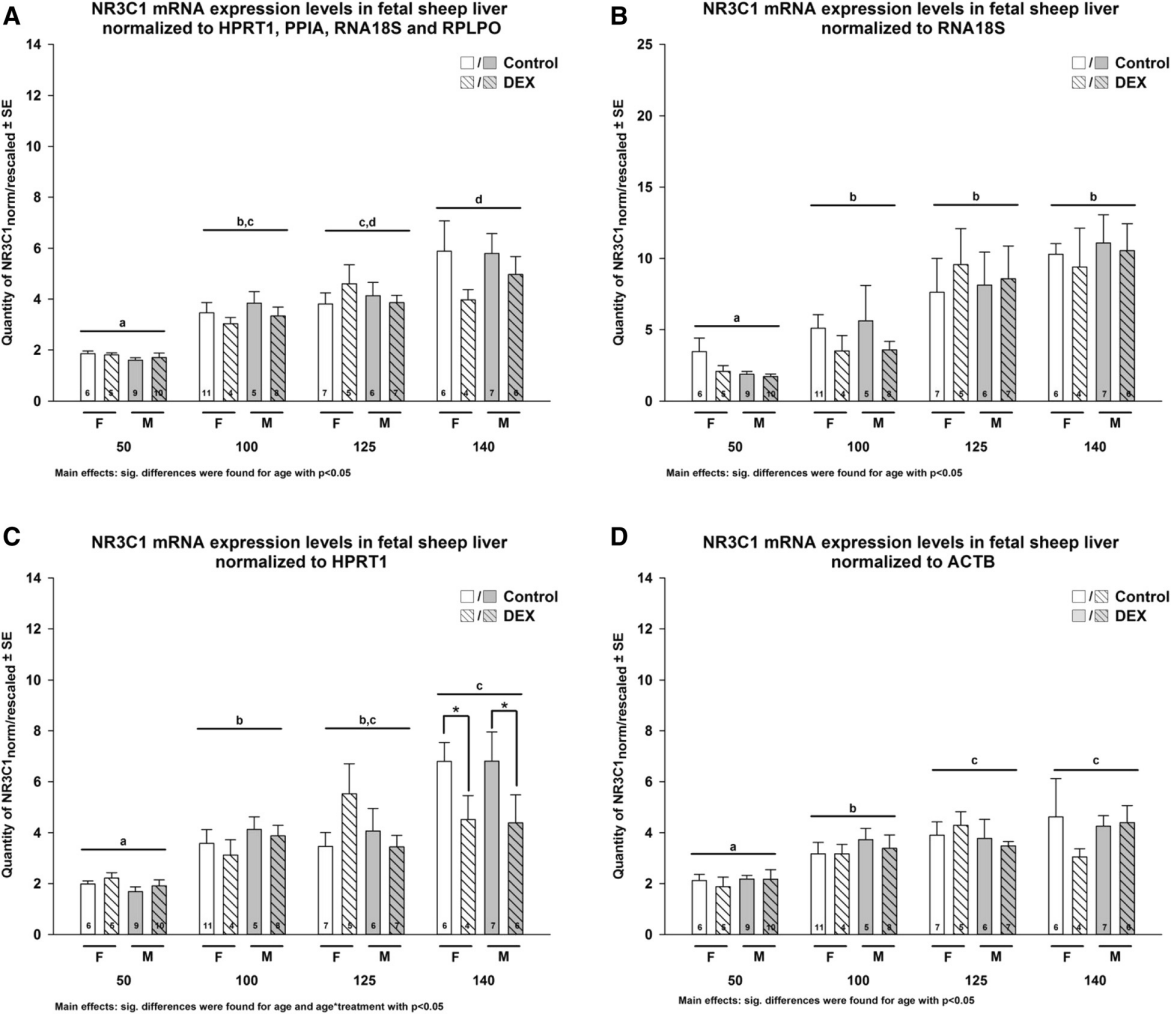
**A-D: Comparison of a target gene mRNA expression normalized with different subsets of internal control genes (ICGs).** *NR3C1* raw RT-qPCR data were normalized to different subsets of ICGs: **(A)** raw RT-qPCR data geometrical mean of *HPRT1*, *PPIA*, *RNA18S*, and *RPLPO*, the most reliable normalization factor as uncovered by the use of geNorm; **(B)** “flat gene” = RNA18S and **(C)** “flat gene” = *HPRT1;* and **(D)** *ACTB* as the least reliable ICGs as uncovered by using geNorm (besides being among the most popular used ICG). Data were analyzed by MANOVA with treatment, gender and day of gestation as main effect, followed by a pairwise comparison (Holm’s Sidak) when main effects were p < 0.05. Different letters indicate significant differences in day of gestation; stars indicate significant differences in DEX treatment. The final data were obtained by rescaled normalized expression: Q_normalized/rescaled_ = (Q_sample_/NF_sample_)/Min (Q_sample_/NF_sample_) (geNorm v3.5 manual [51]).

We have compared the results of normalizing RT-qPCR data of glucocorticoid receptor (*NR3C1*), used as target gene, using the geometrical mean of the 4 best ICGs as indicated by approach 2 (Figure 4A), the two best ICGs (or the more “flat” ICGs *HPRT* and *RNA18S*) indicated by the approach 1 (Figure 4B and 4C) and *ACTB*, the ICG with the lowest average expression stability among the one tested but also one of the most used ICGs in literature (Figure 4D). The statistical differences observed between comparisons on the quantity of *NR3C1* mRNA expression levels obviously differed between subsets of ICGs used for normalization. For example, *NR3C1* mRNA expression significantly increased between 50 and 100dG and further increased between 100 and 140dG when normalized by the geometrical mean of *HPRT1*, *PPIA*, *RNA18S* and *RPLPO* (Figure 4A). However, when normalized only to *RNA18S*, the increase between 100 and 140dG was not significant (Figure 4B). Normalizing to *ACTB* resulted in a significant increase of *NR3C1* mRNA expression between 50 and 125dG (Figure 4D). Normalizing to *HPRT1*, the ICG with the least time effect on the raw RT-qPCR data resulted in a significant reduction of the quantity of *NR3C1* mRNA expression levels at 140dG in DEX compared to controls (Figure 4C), which was not significant when normalized to the other subsets of ICGs (Figure 4A, B, D).

### Dilution effect and ICGs

**Figure 5:**
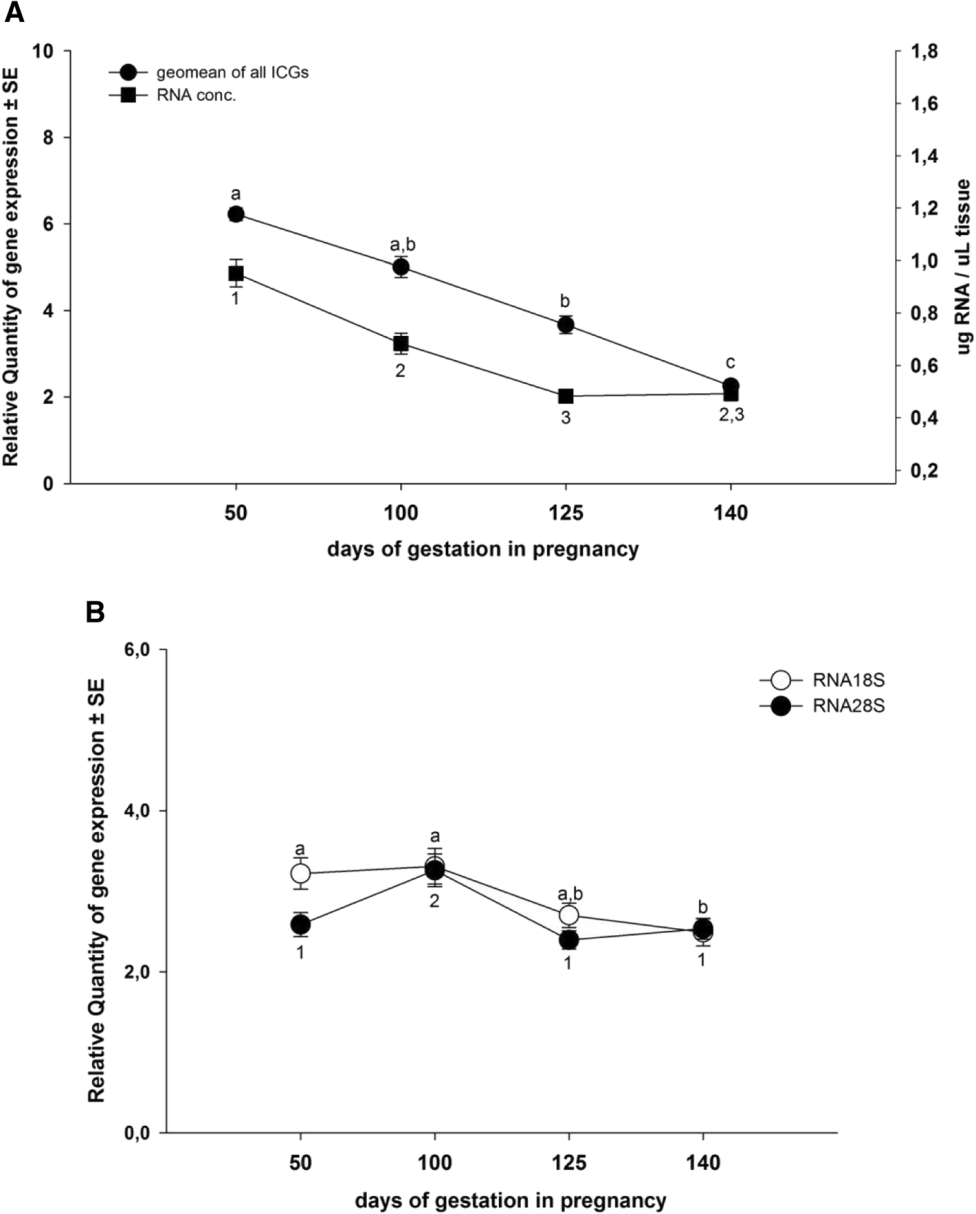
**Pattern of total RNA and raw RT-qPCR data of ICGs and rRNA through gestation. A)** Total RNA concentration (μg/μg tissue) and geometrical average expression of raw RT-qPCR data of all ICGs at four time points during gestation; samples included: 50dG n = 30, 100dG n = 28, 125dG n = 25 and 140dG n = 23. **B)** Pattern of raw RT-qPCR data of *RNA18S* and *RNA28S* during gestation; samples included: 50dG n = 30, 100dG n = 28, 125dG n = 25 and 140dG n = 23. Kruskal-Wallis One Way Analysis of Variance on Ranks (followed by Dunn's test): different letters indicate significant (p < 0.05) differences of ICGs and *RNA18S* and different numbers indicate significance differences in the RNA concentration and *RNA28S* across gestation.

The total RNA was significantly affected by gestation similar to mean raw RT-qPCR data of all ICGs tested (Figure 5A). For this, it is not surprising that we have observed a significant positive correlation between RNA concentration and raw RT-qPCR data for all ICGs tested with exception of *HPRT1*, where the P-value of correlation was 0.06 (Table 2). The raw RT-qPCR data between all ICGs (with exception of *RNA18S*) and insulin-like growth factor 1 (*IGF1*) and glucose 6 phosphatase (*G6PC*) were negatively correlated (Table 2). The raw RT-qPCR data of very abundant expressed ribosomal gene *RNA18S* had the least correlations with the liver-specific genes and total RNA, while raw RT-qPCR data of *RNA28S* did not have any correlation (Table 2). This was mostly due to the very limited effect of gestation on the raw RT-qPCR data (Figure 5B).

## Discussion

Reliability and accuracy of target gene expression levels in RT-qPCR greatly depends on the selection of ICGs; therefore a set of appropriate ICGs must be determined. An ideal ICG for our experiment would be stably expressed under all conditions, i.e., is independent of gestational age, gender or treatment. The previously reported ICGs used for RT-qPCR normalization in fetal sheep liver lacked empirical data demonstrating suitability where many studies have used a single ICG [32,35,52], and the stability of ICGs was not measured or reported.

Various strategies have been applied to normalize for the amount of starting material, differences in tissues, enzymatic efficiencies or overall transcriptional activity. The traditional way of analyzing the accuracy of ICGs is to pick out one or two widely reported ICGs and to test whether experimental conditions affect ICG genes expression levels. Our present study analyzed the effect of gender and DEX treatment at four different gestational time points in fetal liver in six commonly used ICGs in sheep. Significant gestation effect was observed in all measured ICGs. A similar association was found in bovine mammary tissue, in which ICG expression levels significantly changed during the whole lactation [1]. Despite being all ICGs tested affected by gestation, *RNA18S* and *HPRT1* were the least affected. Others have reported changes in expression of many target genes in fetal sheep after GC treatment [32,35,53]. However, in the present study, only raw RT-qPCR data of *TUBB* in females was affected by DEX treatment. Based on the above data and using the criteria of the classical approach (i.e., no effect in expression by the studied condition) we should have rejected all tested ICGs as being unreliable.

Although statistical effects of gestational age for all the ICGs tested were observed in our study, this did not exclude the possibility of a reasonable fit for these genes as ICGs, as indicated in other studies [1,19]. The absence of a statistical effect on potential ICGs is not an essential condition to consider them reliable for normalization and, oppositely, the use of such criteria to select proper ICGs is a serious limitation, especially in experiments dealing with temporal transcriptomics during differentiation [1,19,21]. In fact, the classical approach has two major limitations for selection of reliable ICGs: 1) relying exclusively on the lack of differences between means does not account for the sample-specific variation (i.e., the normalization is performed per each single sample and the use of the means does not account for that specific variation); 2) cannot account for the dilution effect (see below). Very often associated with such an approach there is also the use of a single ICG, which presents several limitations in itself [2].

In order to overcome such limitations, in the current study, we relying on algorithm, particularly geNorm, to evaluate the most stable ICGs and acquire accurate data on the optimal number of ICGs that should be used for normalization [2]. Contrary to the use of the classical approach, geNorm analysis of pairwise variation among the 6 evaluated ICGs in the present study revealed that most of the ICGs tested can be considered reliable and that the use of the geometrical mean of 4 ICGs (*PPIA*, *HPRT1*, *RNA18S* and *RPLPO*) can provide a robust normalization.

The effect of an inappropriate set of ICGs for normalization of a target gene was demonstrated by normalizing the RT-qPCR data of a target gene (*NR3C1*) using the two ICG with the lowest time effect (i.e., the most ‘flat’ genes), i.e., *RNA18S* and *HPRT1* or the 4 most stable ICGs indicated by geNorm analysis among the pool of ICG candidates chosen (geometrical mean of *HPRT1*, *PPIA*, *RNA18S* and *RPLPO*). Despite the fact that all the tested genes had a similar pattern (Figure 1), the analysis revealed obvious differences in the statistical results of the normalized target gene with the best subset of ICGs or the use of only one of the genes least affected by time (*RNA18S* and *HPRT1*). The difference was even more pronounced when the normalization was performed by the very commonly used *ACTB* (Figure 4). This comparison clearly confirmed the limitation of using the classical approach to select ICGs or the use of previously published ICGs without validation using specific algorithms.

The results from the use of geNorm (approach 2) contradict the results from the classical approach (approach 1). In approach 1 all the tested ICGs would have been rejected based on the fact that the raw RT-qPCR data were significantly affected by gestation and/or DEX treatment. All the tested ICGs had a decrease in raw RT-qPCR data through gestation (Figure 1). How is it possible that ICGs can be both deemed to be reliable and have the raw RT-qPCR data significantly affected by a condition (by definition an ICG should not be affected by any condition)? In a previous study in bovine mammary tissue from pregnancy to end of subsequent lactation it was observed a similar pattern of ICGs [1]. In that study it was demonstrated that this pattern is an artifact and consequence of a dilution effect due to equal amount of total RNA is used for RT-qPCR between samples concomitant to a very large expression of few abundant mammary-specific genes with a consequent relative decrease in abundance of the stably expressed genes. In such a case the apparent decrease in raw expression of stably expressed genes is due to the decrease of proportional abundance of their mRNA among all RNAs in the sample and not to active transcription repression.

Based on the similarity between the previous study in bovine mammary tissue and the present study, we have assessed if a dilution effect could have been the cause of the observed reduction of raw RT-qPCR data during gestation (Figure 1; Additional file 1: Figure S1). For this, and similarly to the previous study [1], we have evaluated if a correlation existed between raw RT-qPCR data of ICGs and total RNA concentration. We have also measured the expression of *RNA28S*, the most abundant RNA, and correlated with ICGs. In addition, we have run a correlation with raw RT-qPCR data of the liver-specific genes *IGF1* [54] and *G6PC* [55]. Contrary to the previous study [1], in the present study a significant positive correlation was observed among the raw RT-qPCR data of the tested ICGs and the amount of RNA per μg of tissue. As for the ICGs tested the abundance of RNA decreased through time (Figure 5A). In the same time period the measured amount of rRNA, which is known to make up the majority of total RNA, had a minor change through the differentiation, although tended to decrease. Most of the ICGs decreased more than 3-fold, the rRNAs decreased less than 2-fold, while the total RNA decreased 2-fold (Figure 5A and 5B). Despite the observed decrease through time and correlations, the data are uncoupled. In fact, the total RNA can be considered an absolute value while the values for ICGs and the rRNA are relative. This uncoupling (or lack of direct relationship) is due to the fact that the starting amount of total RNA was equal between all samples (see material and methods) despite the change in total RNA. Therefore, the apparent large decrease in raw RT-qPCR of ICGs and, even with lower magnitude, of the rRNA, strongly support that something else composing the total RNA must have increased dramatically during differentiation and was not measured in the present experiment.

The above observations allow us to conclude that at the beginning of pregnancy there was an overall higher RNA expression leading to higher total RNA concentrations compared to the end of pregnancy. The expression of RNA includes not only mRNA but all RNA species. A similar observed decrease in most of mRNA has been previously reported during the differentiation of human hepatocytes (HepaRG) *in vitro* where a decrease in expression of most of the genes was observed [56]. Therefore, there was an active depression of transcription during differentiation, likely driven by epigenetic specialization [57]. Based on the negative correlations of total RNA and ICGs with liver specific genes, we postulate that there was a large increase in liver specific genes, likely very abundant, such as albumin and fatty acid binding protein 1. This large increase of abundant liver-specific gene can have caused the dilution of other genes, especially the genes with a constant copy number/cells, such is the case for the tested ICGs. Increase in liver-specific abundant genes was also previous observed [56] and are known to be under active transcriptional control by specific liver-specific transcription regulators [58].

A dilution of stably expressed genes in the present experiment can explain the observed apparent, but likely misleading, decrease in raw RT-qPCR data of ICGs (Figure 1 and Additional file 1: Figure S1). Not accounting for such effect, would bias the final normalization of RT-qPCR data of target genes. In our case if a target gene that has an increase in mRNA copy/cell is subject to the dilution effect and it probably appear as having a lower or no change through time if it is normalized by a “flat” gene (i.e., not subjected to the dilution, but, for this has in reality an up-regulation in copy mRNA/cell that is inversely proportional to the dilution). The normalization of RT-qPCR data of that same target gene by using reference genes that proportionally follow the dilution can correct for that dilution revealing the true pattern of the target gene.

The above interpretation of the data has a caveat. If the total mRNA is changing through differentiation this means that there is an overall increase or decrease in overall transcription, as also suggested by previous data from others [56], resulting in an increased or decreased amount of mRNA/cell. This means that the proportion of the 3 types of RNA can change. This observation reveals a limitation on relying only on ICGs for RT-qPCR normalization. The normalization by ICGs is methodologically correct if a relative expression between specific mRNA is considered, but it is not appropriate if an absolute or real expression (i.e., amount of specific mRNA/cell) is considered. The caveat discussed above highlights a shortcoming of using expression of other mRNA to normalize target mRNAs and prompts to find even better approach for RT-qPCR normalization.

## Conclusion

In the present analysis using geNorm we have uncovered that the geometrical mean of *HPRT1*, *PPIA*, *RNA18S* and *RPLPO* can be suitable for calculating the normalization factor to be used with RT-qPCR data in developing fetal sheep liver. The data also indicated that the use of one ICG, even among the list of the 4 more stable ICGs, to normalize a target gene can bias the final results. For this we recommend using the geometric mean of at least the four indicated ICGs as an accurate normalization factor. However, this can be considered reliable only in the present experimental setup. Different experiments, even using the same model, require a separate validation of ICGs, since those are experiment-specific.

In addition, the data clearly indicated that, in order to have a proper relative quantification of target genes, the normalization must be able to account for the dilution effect and also for the variation in total RNA and ratio between RNA species. In the present case the data supported a dilution effect that affected all genes but most visible in stably expressed genes; therefore, proper normalization needs to account for such dilution effect and reliable reference genes should have a decrease through gestation in measured raw RT-qPCR data.

Finally, the present data appear to highlights a limitation of using ICGs for normalization when a change in overall transcription during tissue differentiation is observed, i.e. increase/decrease of mRNA as was suggested by the data in the present experiment. Therefore, even if the proposed normalization can be considered reliable for relative gene expression, the quest for an absolute way to normalize RT-qPCR data is ever more necessary.

## Methods

### Animals and tissues

All procedures were approved by the Animal Experimentation Ethics Committee of the University of Western Australia and/or the Western Australian Department of Agriculture [26].

### Prenatal treatments

Pregnant Merino ewes (Ovis aries) with singleton pregnancies (total n =106 liver samples) of known gestational age were allocated randomly to receive maternal injections of DEX or saline (control). Maternal DEX (Mayne Pharma, Victoria, Australia) injections were given in a dose of 0.14 mg/kg ewe body weight suspended in 2 mL of saline, consisting of four intramuscular injections at 12 h intervals over 48 h between 40 and 41 days of gestation (dG). Control animals received saline injections of a comparable volume (2 mL saline/ewe) [26].

### Tissue collection

Tissue samples were collected at 49–51 (50), 101–103 (100), 125–127 (125), and 140–142 (140) dG. Fetal liver and other major fetal organs were removed, weighed, and collected for use in other studies (e.g., [26]). Right liver lobe (100, 125 and 140dG) and total liver (50dG) biopsies were snap frozen in liquid nitrogen before storage at −80°C.

### Real time PCR protocol

Total liver RNA was extracted using the RNeasy Midi kit (QIAGEN, Clifton Hill, Victoria, Australia) and stored at-80°C until further use. RNA concentration in μg/μg tisse was determined by NanoDrop 1000 Spectrophotometer (average ^260^/_280_ = 2.04 ± 0.08; Thermo Fisher Scientific, Wilmington, U.S.A.) and integrity was analyzed in a selected number of samples with Agilent 2100 BioAnalyzer (RNA 6000 Nano Kit 5067–1511; RIN mean 8.2). Possible genomic DNA contamination was removed from each sample using a DNase treatment (Fermentas, Thermo fisher scientific, Catalog #EN0521), and then RNA sample (1 μg) was reverse transcribed in a 20 μl reaction mixture (iScript cDNA synthesis kit, BIO-RAD, Catalog #170-8890) according to the manufactures manual. DNase treatment and RT reactions were carried out in a Mastercycler (Eppendorf, Germany). For each run a no template control sample containing no RNA was reverse transcribed to provide a negative control for real time PCR applications. For RT-qPCR, primer pairs for sheep (Table 3) were either designed using Primer 6.0 (*TUBB, PPIA, ACTB, RNA28S*) or were previously reported (*RPLP0* [59], *RNA18S* [26], *HPRT1* [60], *NR3C1* [26], *IGF1* [59], *G6PC* [61]). All primer sequences have been tested and verified with fluorescent color band sequencing (PCR purification kit #83050, Seqlab, Sequence Laboratories Göttingen, Germany).

**Table 3:** **Primer information for candidate reference genes and rRNA in fetal sheep liver**

**Gene symbol**	**Primer sequences (5’ → 3’)**	**Product size**	Efficiency*	**Accession No.**	**Function and reference**
***RPLP0***	F:CAA CCC TGA AGT GCT TGA CAT	227 bp	95.3%	NM_001012682	Protein metabolism and modification [59,60]
	R: AGG CAG ATG GAT CAG CCA				
***TUBB***	F:GGT CCT GGA TGT GGT TCG GAA G	223 bp	99.78%	GQ338157	Member of a small family of globular proteins [62]
	R:GAC GGA GAG GGT GGC ATT GTA G				
***RNA18S***	F: GCT ACC ACA TCC AAG GAA GG	244 bp	95.62%	NR_036642.1	Recognition role, involved in correct positioning of the mRNA and tRNA [26,51]
	R: GCT CCC AAG ATC CAA CTA CG				
***ACTB***	F:CAT CGG CAA TGA GCG GTT CC	146 bp	96.02%	NM_001009784	Cytoskeletal structural protein [60]
	R:CCG TGT TGG CGT AGA GGT				
***HPRT1***	F:GCT GAG GAT TTG GAG AAG GTG T	94 bp	95.6%	NM_001034035.1	Nucleoside, nucleotide and nucleic acid metabolism [60]
	R:GGC CAC CCA TCT CCT TCA T				
***PPIA***	F:TGT GCC AGG GTG GTG ACT TCA	196 bp	100%	AY251270	Protein metabolism and modification [60]
	R:TGC TTG CCA TCC AAC CAC TCA G				
***NR3C1***	F: ACT GCC CCA AGT GAA AAC AGA	151 bp	95.6%	NM_001114186	glucocorticoid receptor [63]
	R: ATG AAC AGA AAT GGC AGA CAT				
***IGF1***	F: TTG GTG GAT GCT CTC CAG TTC	118 bp	95.1%	NM_001009774.2	Insulin like growth factor type 1 [59]
	R: AGC AGC ACT CAT CCA CGA TTC				
***G6PC***	F: GGA TTC TGG ATC GTG CAA CT	196 bp	100%	EF062861.1	Glucose 6 phosphatase [61]
	R: ATC CAA TGG CGA AAC TGA AC				
***RNA28S***	F: AAC TCT GGT GGA GGT CCG TAG C	115 bp	97.5%	XM_004023057	structural RNA for the large component of eukaryotic cytoplasmic ribosomes, and thus one of the basic components of all eukaryotic cells
	R: GAG GGA AAC TTC GGA GGG AAC C				

*PCR efficiencies were determined using the formula (10^-1/slope^-1) × 100%.

The RT-qPCR analysis was run in triplicates on an ABI 7500 Real Time PCR System (Applied Biosystems, Foster, USA). All measurements were run in a total volume of 10 μl including 5 μl power SYBR green master mix (Applied Biosystems, Foster, USA), 4 μl cDNA, 0.4 μl primer pair in a final concentration of 400 nM and 0.2 μl water. A non template control was included in each RT-qPCR run for each primer. After gradient PCR optimization with a 10 step annealing temperature gradient according to the manufactures protocol (Mastercycler gradient, Eppendorf Germany), all transcripts were amplified using the following cycling conditions: 95°C for 10 min for one cycle and 95°C for 20 sec, 60°C (*RPLPO*, *TUBB*, *RNA18S*, *ACTB*, *HPRT1*, *PPIA, IGF1*, *G6PC* and *RNA28S*) or 58.9°C (*NR3C1*) for 32 sec, and 72°C for 60 sec for 40 cycles (Table 3). Melting-curve analysis (95°C for 15 sec, 60°C for 60 sec, 95°C for 15 sec and 60°C for 15 sec for one cycle) demonstrated a single PCR product in all measurements. Efficiencies of all genes were calculated in the same sample with E(%) = 100x(10^1/slope^ -1) [64] with same ABI 7500 PCR program and deemed to be similar to each other (range from 95.3% to 100%; see Table 3).

### Quantification and ICG stability evaluation

The geomean of threshold cycle (Ct) values from the 7500 system software SDS software version 1.4 (Applied Biosystems, Foster, USA) were exported to Microsoft Excel. The quantity of each ICG was calculated by Q = Efficiency^ΔCt^ where ΔCt = Ct min-Ct sample for each gene. Statistical analyses were performed by using SPSS 20 statistical software (SPSS Inc., Chicago, USA). Data was tested for normal distribution and equal variance (Levene test, p > 0.05). Data that were not normally distributed (*RPLPO*, *TUBB*, *ACTB*, *HPRT1*, *PPIA*, *NR3C1*) were log transformed to achieve normality. To determine treatment, day of gestation, and gender effects as well an interaction between them on raw or normalized RT-qPCR data, a MANOVA with treatment, gender and day of gestation as factors, followed by a pairwise comparison (Holm’s Sidak) when main effects were p < 0.05 was performed. Main effects are indicated in the figure legend; post hoc p-values (Holm-Sidak) are indicated in figures. Data are presented as mean ± S.E.M. Statistical significance was accepted for values p < 0.05. The relationship between the average expression of ICGs and RNA concentration (ug/ug tissue) was analyzed with the Pearson correlation.

To determine the reliability of ICGs using the geNorm analysis the average expression stability values (M) of 6 ICG genes in all samples were analyzed with geNorm Visual basic application (V 3.5, Biogazelle NV, Zwijnaarde, Belgium) according to the manufactures manual and the procedures described by Vandesompele *et al.* [2] with respect to days of gestation, gender and treatment. The raw RT-qPCR data were then uploaded into geNorm, Bestkeeper, or Normfinder and ICGs ranked based on algorithm-specific values. For geNorm the ICGs were ranked based on the M value, which refers to the constancy of the expression ratio between two genes among all samples tested [1]. At least two ICGs are required for stability analysis and subsequent M values are calculated prior to the stepwise exclusion of the least stable ICGs. The lowest M value indicates the pair of ICGs with the most stable expression.

In order to determine the minimum number of ICGs which has to be used to obtain a reliable normalization factor (NF), geNorm calculates the pairwise variation Vn/n + 1 between NFn and NFn + 1 from the NF calculated using the two most stable genes (i.e., with lower M) and the NF calculated using the additional more stable gene (i.e., n + 1) and so on until the stepwise inclusion of subsequent less stable ICGs has no significant contribution to the newly calculated NF (Figure 2). For example, V2/3 shows the variation of the NF of two genes in relation to three genes. geNorm suggests a cut-off for the pairwise variation V of ≤ 0.15. A relatively large decrease in pairwise variation V indicates that the added gene has a significant effect on the final NF and should be included in the calculation of NF. However, the proposed cut off at ≤0.15 of the V is considered to be indicative and the lower the V the higher the reliability of NF [2]. Ideally the NF should be calculated by the geometrical mean of the combination of ICGs with the lower V-value. According to geNorm protocol the use of the three best ICGs is in most cases a valid normalization strategy, and results are much more accurate and reliable compared to the use of only one ICG [2].

It is known, that the geNorm approach has the problem that it tends to select genes with similar expression profiles [65]. Therefore, the pairwise comparison approach of geNorm to determine ICG stability is highly biased by potential co-regulation between selected ICGs [1,65]. The potential ICGs should not be regulated through common upstream effectors or should not directly regulate each other [22]. We therefore analyzed in a post-run check potential co-regulation between the 6 potential ICGs with Ingenuity Pathway Analysis web-based software, (Ingenuity System accessed 12.12.2012, default settings were used, one relationship step was analyzed; www.ingenuity.com, Version 14400082, Build 192063, Redwood City, CA). The software allows the discovery, exploration and visualization of gene interactions and co-regulations. Networks are generated relying on known published relationships among human, mouse and rat genes. Briefly, the network analysis was performed using only relationship types including “expression”, “transcription” and “any direction”. The “Build-Grow” option was used and all the up-stream transcription factors of our ICGs were added. The following options were selected: Interactions = “direct” and “indirect”; Grow out”All the molecules” that are “Upstream of the selected molecules” and molecules were limited to “Use Ingenuity Knowledge Base”; Relationship Types = “expression” and “transcription”; Molecule Types = “ligand-dependent nuclear receptor” and “transcription regulator”. All the other options were left as default. Once all the upstream molecules had been added by IPA, we manually deleted all the transcription factors that had only one relationship with our ICGs.

Quantities of the exemplary target gene (*NC3R1*) was calculated by: Q = efficiency^(ct min - ct sample), rescaled normalized expression levels Q_normalized/rescaled_ = (Q_sample_/NF_sample_)/Min(Q_sample_/NF_sample_) (geNorm v3.5 manual [66]), where NF was calculated using the number of most reliable ICGs as uncovered by geNorm.

## Additional file

Additional file 1: Figure S1. Comparison of relative quantities of 6 ICGs across gestation. Kruskal-Wallis One Way Analysis of Variance on Ranks: different numbers indicate significant differences in RQ of ICGs (clour coded) across gestation with p<0.05. Relative Quantity of gene expression = efficiency^(ct min - ct sample)/ RQmin.

## Abbreviations

RNA18S: 18S ribosomal rRNA
RNA28S: 28S ribosomal rRNA
ACTB: Beta actin
DEX: Dexamethasone
FOS: FBJ murine osteosarcoma viral
GC: Glucocorticoid
NR3C1: Glucocorticoid receptor
HPRT1: Hypoxanthine phosphoribosyltransferase 1
ICG: Internal control gene
M: Average expression stability value
MYC: v-myc cytelomatosis viral oncogene homolog
MYCN: v-myc myelocytomatosis viral
NF: Normalization factor
PPIA: Peptidylprolyl isomerase A
RPLPO: Ribosomal protein, large, P0
RT-qPCR: Reverse transcription quantitative polymerase chain reaction
TP53: Tumor protein p53
TUBB: Tubulin beta
V: Defines the pairwise variation between sequential NFs
