# Supplementary material for: The dilution effect and the importance of selecting the right internal control genes for RT-qPCR: a paradigmatic approach in fetal sheep
Source: BMC Res Notes. 2015 Feb 27;8:58. doi: 10.1186/s13104-015-0973-7 (PMC4352295; doi:10.1186/s13104-015-0973-7)
Supplement: Additional file 1: Figure S1. — Comparison of relative quantities of 6 ICGs across gestation. Kruskal-Wallis One Way Analysis of Variance on Ranks: different numbers indicate significant differences in RQ of ICGs (clour coded) across gestation with p<0.05. Relative Quantity of gene expression = efficiency^(ct min - ct sample)/ RQmin. [file 13104_2015_973_MOESM1_ESM.pdf]

Supplement:

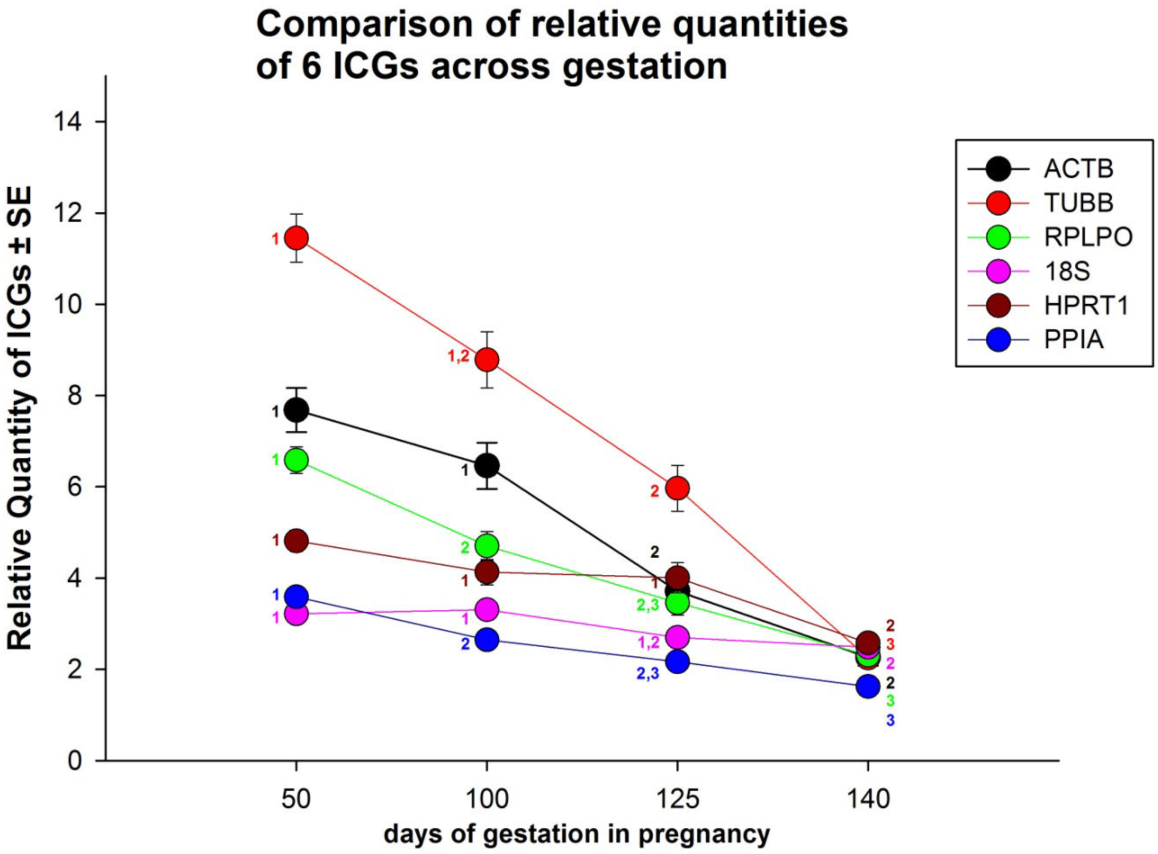

Kruskal-Wallis One Way Analysis of Variance on Ranks: different numbers indicate significant differences in RQ of ICGs (clour coded) across gestation with p<0.05.
Relative Quantity of gene expression = efficiency^(ct min - ct sample) : RQmin
